# Can Thyrotropin, Tri-Iodothyronine, and Thyroxine Hormones be Predictors of Cancer in Thyroid Lesions?

**DOI:** 10.7759/cureus.32422

**Published:** 2022-12-12

**Authors:** Mohamed O Khider, Caroline Ayad, Awadia G Suliman, Sultan A Alshoabi, Moawia Gameraddin, Maisa Elzaki, Walaa Alsharif, Mohammed Arafat, Ahmed Alali, Khalil Abu Odeh

**Affiliations:** 1 Department of Medical Imaging, King Fahad Specialist Hospital, Dammam, SAU; 2 Department of Diagnostic Radiology, SUST, Khartoum, SDN; 3 Department of Diagnostic Radiology Technology, College of Applied Medical Sciences, Taibah University, Al-Madinah Al-Munawarah, SAU; 4 Faculty of Radiological Sciences and Medical Imaging, Alzaiem Alazhari University, Khartoum, SDN

**Keywords:** malignancy, triiodothyronine (t3), thyroxine (t4), thyroid-stimulating hormone (tsh), thyroid nodules

## Abstract

Background

Thyroid nodules are a common medical problem worldwide. This study aims to investigate and elucidate the relationship between thyroid-stimulating hormone (TSH), thyroxine (T4), and triiodothyronine (T3), and malignant thyroid nodules.

Methods

This prospective cross-sectional study was conducted at a public specialist hospital in Saudi Arabia from February 2020 to February 2021. All thyroid nodules were scanned using ultrasound imaging, and the largest diameter was measured for each and classified according to the American College of Radiology (ACR) Thyroid Imaging Reporting and Data System (TIRADS) classification system. Thyroid function tests TSH, T3, and T4 were measured. Definitive diagnoses of thyroid nodules were given based on cytology. A one-way analysis of variance (ANOVA) test was used to compare means, and cross-tabulation was used to correlate the variables in the study.

Results

A total of 222 patients participated in this study; 23.42% were male and 76.57% were females. The mean age was 44.73 ± 13.31 years (range: 18 to 85 years). The percentage of malignancy was 20.6%, 36.3%, and 91.2% in TIRADS 3, TIRADS 4, and TIRADS 5, respectively. A weak positive linear relationship was noted between nodule size and TSH (R^2^= 0.012). The study demonstrates that TSH increases in malignant nodules more than in benign nodules, while T4 and T3 are decreased in malignant nodules.

Conclusion

The level of TSH increases in patients with malignant thyroid nodules more than in benign nodules, which can be used as a predictor of malignancy, while T4 and T3 reduced in malignant nodules with an ambiguous relationship.

## Introduction

The American Thyroid Association (ATA) defines the thyroid nodule as a disc lesion within the thyroid gland, which is radiologically distinct from the thyroid parenchyma. Thyroid nodules are a common medical problem with the prevalence as palpable nodules of approximately 5% in females and 1% in males living in iodine-sufficient areas of the world. However, high-resolution ultrasound imaging can detect thyroid nodules in up to 68% of people, with higher frequencies in females and the elderly [1]. Thyroid nodules are clinically crucial for several reasons, primarily because of their malignant potential in 4-6.5%, largely independent of the nodule size [2].

Fine needle aspiration (FNA) biopsy is the most reliable method for diagnosing thyroid malignancy and determining nodules that require surgical intervention [2,3]. A diagnostic approach using ultrasound imaging and, when indicated, FNA biopsy and molecular testing facilitates a risk-based personalized protocol that promotes high-quality healthcare and minimizes unnecessary testing and cost [3]. Ultrasound imaging and serum thyroid-stimulating hormone (TSH) are pivotal in evaluating thyroid nodules as they provide good information about features suspicious for malignancy and nodule functionality. Serum TSH should be measured in all thyroid nodule patients with an increased risk of thyroid malignancy [4]. Thyroxine (T4) and triiodothyronine (T3) are the main hormones made by the thyroid gland. Measuring levels of these hormones provide a sense of thyroid function. However, the average levels of T4 are similar in malignant and benign thyroid nodules [5].

Thyroid nodules represent a diagnostic challenge because of the need to determine thyroid malignancy to avoid unnecessary surgical intervention in benign thyroid nodules. Basic and simple clinical measurements, such as TSH, T4, and T3, are essential to understand the nature of indeterminate thyroid nodules and differentiating malignant ones. A discrepancy regarding TSH, T4, and T3 levels in malignant thyroid nodules was found in the literature [6,7]. Therefore, this study was designed to investigate the relationship between TSH, T4, and T3, and malignant thyroid nodules, solve this argument, and elucidate the role of these hormones as a predictor of malignancy in a thyroid nodule.

## Materials and methods

Ethical consideration

The Institutional Review Board granted ethical approval to King Fahad Specialist Hospital, Dammam, Saudi Arabia (Reference Number: RAD0317). Informed consent was obtained from each participant enrolled in this study. The confidentiality of the participants was assured during and after this study.

Study design and participants

A prospective cross-sectional study was conducted on 222 patients with suspected thyroid nodules at a public specialist hospital in Saudi Arabia from February 2020 to February 2021. All patients were scanned using General Electric (GE) ultrasound OGIQ E10, E9, and S8 ultrasound machines with high-frequency probes (9 and 15 MHz) for data collection. Ultrasound scanning of the gland was performed with the patient lying supine and the neck stretched. The thyroid gland and surrounding neck tissue scan were performed in longitudinal and transverse planes. In a transverse scan at the midline with the probe swept superior and inferior, the isthmus was evaluated and measured in anteroposterior dimension. In a longitudinal approach, both lobes were scanned from the middle to medial and lateral aspects. After that, the transducer was rotated counterclockwise to capture transverse images at the superior and inferior midpoints of each lobe. The right and left lobes' length, width, thickness, and volume were completely estimated. The evaluation of thyroid nodules included their location (left lobe, right lobe, or isthmus), maximum diameter, composition, echogenicity, shape, border, and presence of echogenic foci. Each nodule was classified based on composition, echogenicity, shape, border, and echogenic foci (TIRADS levels 1-6). Definitive diagnoses of thyroid nodules were given based on cytology. Thyroid function tests, TSH, T3, and T4, were measured. In all patients, the thyroid nodules' largest diameter was measured, and all nodules were classified according to the Thyroid Imaging Reporting and Data System (TIRADS 2017) classification system of the American College of Radiology (ACR) using an ultrasound scan. Mild, moderate, and high suspicious nodules (ACR-TIRADS categories 3, 4, and 5) were included in the study sample, while TIRADS categories 1 and 2 and any patients who refused the FNA were eliminated. The researcher in this study specifically designed and used a data collection sheet. This data collection sheet included demographic data, ACR TIRADS classification of thyroid nodules, thyroid function tests, and FNA results.

Data analysis

The collected data were analyzed using the SPSS version 23 (IBM Corp., Armonk, NY, USA); cross-tabulation was utilized to correlate the study variables. Means ± standard deviations were used to describe continuous variables. Frequencies and percentages were used to describe categorical values. The means of hormone measurements in benign and malignant thyroid nodules were compared using a one-way analysis of variance (ANOVA) test. A p-value of ≤0.05 was deemed statistically significant.

## Results

A total of 222 patients participated in this study. The demographic data showed that 23.42% were males and 76.57% were females. The mean age was 44.73 ± 13.31 years (range: 18 to 85 years). There was no significant difference found in the mean age among patients with benign and malignant thyroid nodules (45.8± 13.2 versus 43.5±13.3%); females were affected by thyroid nodules more than the males (76.57% [n=170] versus 23.42% [n=52]). The study results revealed that the most affected age group by malignancy was 18-40 years and then 41-60 years. The percentage of thyroid malignancy was 35.58% in females and 13.06% in males, and no significant difference was noted (p > 0.05). The prevalence of malignant nodules was higher than benign nodules in males, while the prevalence of benign nodules was more in females than malignant nodules (Table 1).

**Table 1:** Demographic data and nodule size in patients with thyroid nodules.

Demographic characteristic	Benign	Malignant	Total	P-value
Age (years)	45.8± 13.2 (22-85 years)	43.5±13.3 (18-76 years)	44.43±13.31 (18-85 years)	0.195
Maximum diameter (cm)	3.02± 1.13 (1.02-7 cm)	2.90±1.70 (1-11 cm)	2.97±1.44 (1-11 cm)	0.539
Gender				
Male	23 (10.36%)	29(13.06)	52 (23.42%)	0.155
Female	91 (40.99)	79 (35.58%)	170 (76.57%)	
Age groups				
18-40 years	48 (21.62%)	49 (22.07)	97 (43.69%)	0.774
41-60 years	50 (22.52)	47 (21.17)	97 (43.69%)	
>60 years	16 (7.2%)	12 (5.41%)	28 (12.61%)	
Total	114 (51.35%)	108 (46.65%)	222 (100%)	

According to histopathology results, a significant correlation was found between an increased TIRADS category and growing malignancy. The percentage of malignancy was 20.6%, 36.3%, and 91.2% in TIRADS 3, TIRADS 4, and TIRADS 5, respectively. The presence of enlarged lymph nodes was 0.00, 5.5%, and 33.82% in TIRADS 3, TIRADS 4, and TIRADS 5, respectively (Table 2).

**Table 2:** Correlation between TIRADS scores with FNA results in the presence of lymph nodes TIRADS, Thyroid Imaging Reporting and Data System; FNA, fine needle aspiration

Characteristics	TIRADS 3	TIRADS 4	TIRADS 5	Total	P-value
Benign	50 (79.4%)	58 (63.7%)	6 (8.8%)	114	<0.001
Malignant	13 (20.6%)	33 (36.3%)	62 (91.2%)	108	
Presence of lymph nodes					
Yes	0 (0.00%)	5 (5.5%)	23 (33.82%)	28	<0.001
No	63 (100%)	86 (94.5%)	45 (66.18%)	194	
Total	63	91	68	222	

A significant increase was observed in TSH in TIRADS 5 nodules compared to TIRADS 3 and TIRADS 4 (p<0.05). There was no significant differences in T3 and T4 in TIRADS 3, TIRADS 4, and TIRADS 5 (p>0.05). Concerning T3, it was slightly elevated in TIRADS 5 compared to both TIRADS 3 and TIRADS 4, while T4 reduced gradually as TIRADS level increased (Table 3).

**Table 3:** Correlation between TIRADS scores and thyroid hormones TIRADS, Thyroid Imaging Reporting and Data System

Thyroid hormones	Normal range	TIRADS levels			P-value
		TIRADS 3	TIRADS 4	TIRADS 5	
TSH (mIU/L)	0.4-4 mIU/L	4.76±2.50	3.86±2.37	5.04±2.90	0.012
T3 (pmol/L)	3.1-6.8 pmol/L	3.75±0.72	3.80±0.79	3.86±0.67	0.708
T4 (pmol/L)	12-22 pmol/L	12.48±3.12	12.27±3.03	11.78±2.57	0.364

The mean level of TSH was higher in patients with malignant nodules than benign ones, while T3 and T4 reduced in patients with malignant nodules (Table 4).

**Table 4:** Comparison of TSH, T3, and T4 hormones with FNA results. FNA, fine needle aspiration

Thyroid hormones	FNA results		P-value
	Benign	Malignant	
TSH	4.36±2.52 (0.22-12)	4.61±2.73 (0.39-14)	0.473
T3	3.88±0.81(2.6-5.7)	3.73±0.64 (2.7-5.6)	0.125
T4	12.55±3.04 (9-19)	11.79±2.76 (8-19)	0.051

**Figure 1:**
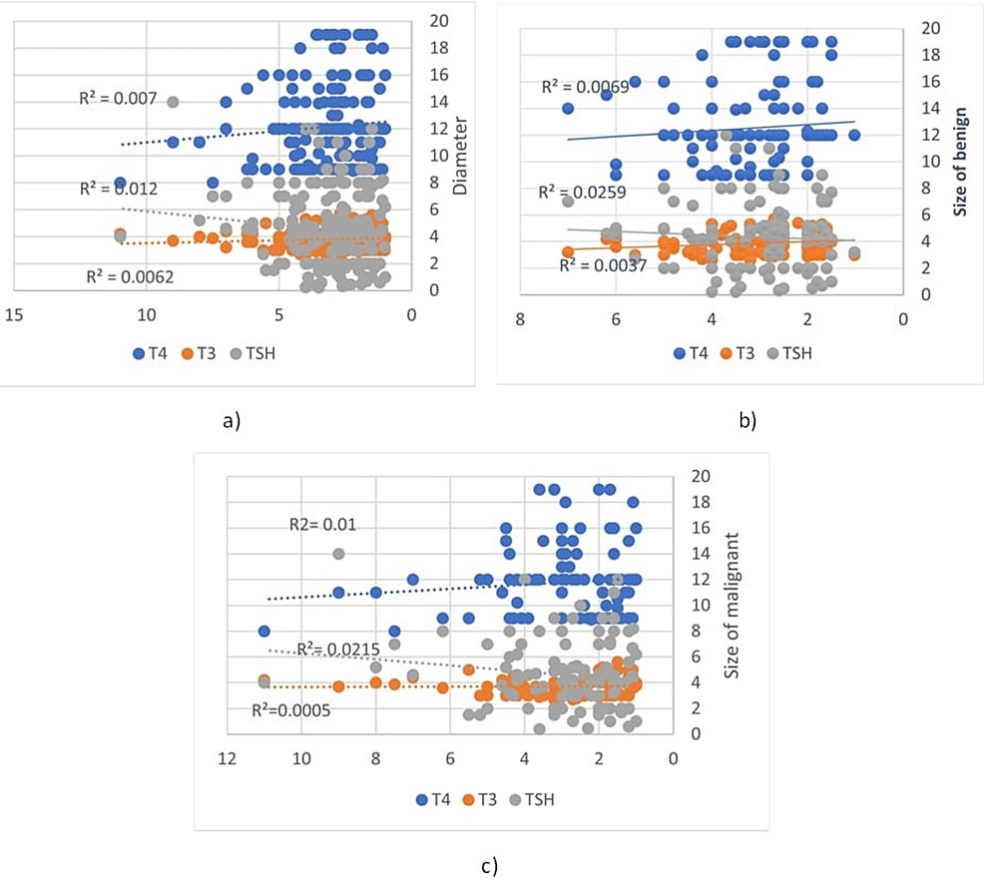
(a) A linear relationship between nodule size and TSH, T3, and T4 hormones. (b) A linear relationship between nodule size and TSH, T3, and T4 hormones in benign nodules. (c) The relationship between nodule size and TSH, T3, and T4 in malignant nodules.

**Figure 2:**
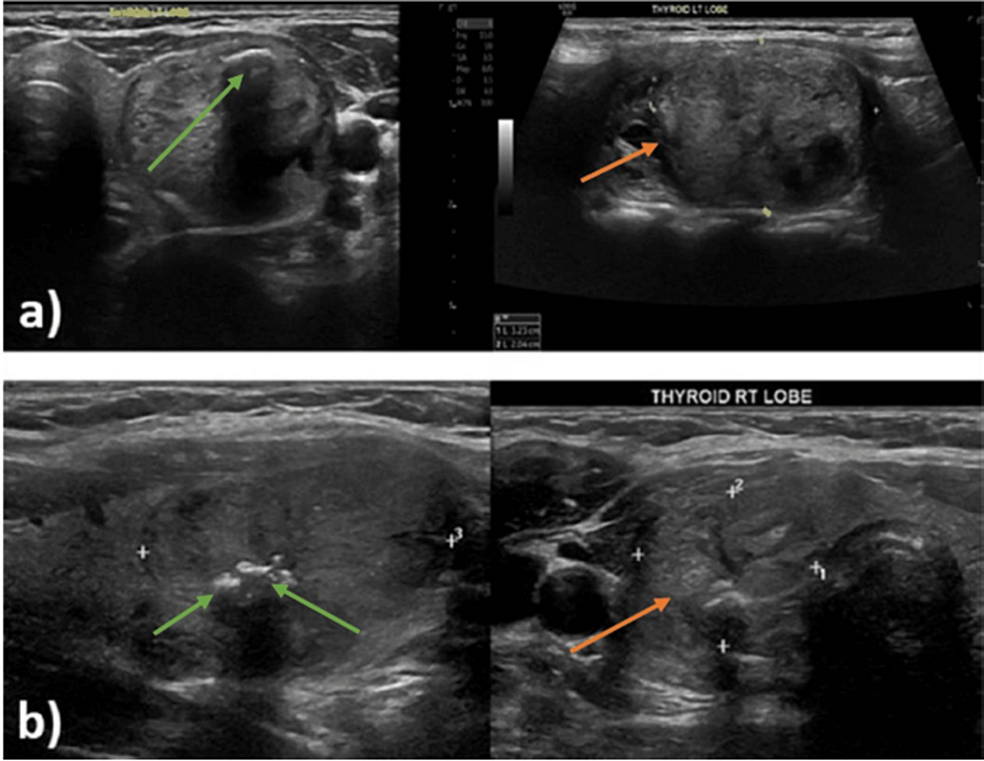
Thyroid sonograms. (a) A 55-year-old female with a well-circumscribed isoechoic left thyroid nodule (orange arrow) measuring 32.3 mm in the largest diameter with a focus of macrocalcification (green arrow), confirmed as a benign thyroid nodule on FNA. (b) A 27-year-old female with an ill-circumscribed isoechoic right thyroid nodule (orange arrow) measuring 30.4 mm in the largest diameter with multiple foci of microcalcification (green arrow), confirmed as a malignant thyroid nodule on FNA. FNA, fine needle aspiration

The results showed an increase in TSH and reduction in both T3 and T4 with increasing thyroid nodule size. A positive linear relationship was noted between nodule size and TSH (R^2^ = 0.012). In contrast, a negative relationship was noted between nodule size and both T3 and T4 (R^2^ = 0.007 for T4 and size and 0.0062 for T3 and size of the nodule (Figure 1a). Despite the weak linear positive relationship between nodule size and TSH, this study demonstrates that TSH increases in malignant nodules more than in benign nodules when the nodule size increases (R^2^ for correlation was 0.0215 in malignant and 0.0037 in benign) (Figures 1b, 1c). Figure 2 shows thyroid sonogram revealing sonographic characteristics of benign and malignant nodules.

Likewise, in logistic regression, the risk of malignancy increased 3.54 odds in the TSH value in the range of 1-2 mIU/L compared to TSH values lesser than 1 mIU/L. On the other hand, the risk of malignancy also increased 2.85 odds in TSH levels more than 2 mIU/L compared to TSH values lesser than than 1 mIU/L, as shown in Table 5.

**Table 5:** Risk of thyroid malignancy associated with different TSH levels

TSH levels (mIU/L)	OR	95% CI	Z statistics
1-2 compared to lesser than 1	3.54	0.85-14.73	1.74
More than 2 compared to lesser than 1	2.85	0.74-10.93	1.53

## Discussion

Thyroid nodules are a common health problem with a significant percentage of malignancy. It represents a diagnostic challenge because of the need to determine thyroid malignancy and avoid unnecessary surgical intervention in benign thyroid nodules. This study was introduced to investigate and elucidate the relationship between TSH, T4, and T3, and malignant thyroid nodules as easily achieved predictors.

The average TSH level and the histopathological diagnosis were shown to differ insignificantly. However, the mean TSH level was higher in patients with malignant thyroid nodules than in those with benign ones, which can be utilized as a predictor of malignancy. Consistently, higher blood TSH levels have been associated with an increased risk of thyroid cancer in individuals with thyroid nodules, according to prior research relevant to this one [8-10]. Our result is in line with a previous study by Fiore et al. They reported that high levels of TSH, even within the normal range, are associated with a higher risk of thyroid malignancy. Lower levels of TSH are associated with a lower risk of papillary thyroid cancer (PTC) [11]. The result of the current study was also consistent with a previous study carried out by Fiore and Vitti [12]. They reported that higher TSH levels are associated with a higher frequency and advanced stage of thyroid cancer. Furthermore, they reported a strong relationship between TSH levels and the risk of PTC. An additional study reported that TSH is a principal hormone for differentiated papillary and follicular thyroid cancers [13]. In contrast, a study found insignificant differences in TSH and type of thyroid carcinomas [14].

The present study found that the level of TSH was significantly elevated in highly suspicious thyroid malignancy (TIRAD5) than in mild suspicious ones (TIRAD3) and more than in benign nodules. Therefore, it can be used as a predictor of malignancy. Baser et al. reported that malignant thyroid nodules and higher suspicious thyroid nodules have higher TSH levels than benign ones [15].

The study found that the risk of malignancy is 2.8-fold in concentration of TSH more than 2 MLU/L related to lower values. This result is consistent with that reported by Golbert et al., who stated that the risk of thyroid malignancy increased 3-folds in patients with TSH ≥ 2.26 than patients with lower TSH levels [8]. Additionally, another study found that the prevalence of malignancy was more in patients with TSH levels ≥ 2.7 MLU/L [16].

It was found that T3 and T4 reduced with the increasing size of thyroid nodules and that they both also decreased in malignant nodules. Regarding T3 hormone, Fitriyani et al. found a significant relationship in thyroid carcinoma, and Jonklaas et al. supported that patients with thyroid malignancy have lower T3 levels than patients with benign nodules [14,17]. In contrast to our findings, Sasson et al. found that low levels of free T4 hormones enhanced the probability that a nodule was benign. [5].

This study identified insignificant relationship between T3 and thyroid malignancy, it was lower values in malignancy which consistent with Jonklaas et al. [17]. Additionally, Rinaldi et al. found lower risk of cancer in patients with low T3 [18]. Although the current study found that T3 and T4 tend to be low in cases of malignancy, the actual relationship between these hormones and malignant thyroid nodules is still a debate, and further studies are needed to resolve this topic.

Limitations of this study

This study has several limitations. Firstly, the thyroid nodules were classified as benign and malignant without mentioning the type and grade of malignancy. Some malignant nodules are more aggressive than others, and thus this may cause more disturbance in thyroid hormone levels than others. Secondly, the T4 and T3 cut-offs are at the upper or lower limits of the normal range, respectively. Further studies are required concerning the relationship between the malignant and benign nodules and T3 and T4 thyroid hormones.

## Conclusions

Based on a sonographic analysis of the thyroid nodules and the laboratory values of thyroid hormones, the level of TSH increases in patients with high suspicious malignant thyroid nodules more than in those with mild suspicious ones and more than in those with benign nodules, which can be used as a predictor of malignancy. Low levels of T3 and T4 are noted in malignant thyroid nodules despite the insignificant relationship.
